# Safety and Efficacy of Single-Session Endoscopic Retrograde Cholangiopancreatography and Laparoscopic Cholecystectomy for the Treatment of Concomitant Gallbladder and Common Bile Duct Stones: A Single-Centre Experience

**DOI:** 10.7759/cureus.113857

**Published:** 2026-08-03

**Authors:** MG Prakash, Yashodhara N, GR Samruddhi, MP Sathvika

**Affiliations:** 1 General Surgery, JJM (Jagadguru Jayadeva Murugarajendra) Medical College, Davanagere, IND

**Keywords:** cbd stenting, cholangitis, choledocholithiasis, cholelithiasis, ercp, laparoscopic cholecystectomy

## Abstract

Background and objectives

Cholelithiasis is a commonly encountered biliary disorder in the Asian subcontinent, with a substantial burden observed in the Indian population. A significant proportion of patients admitted with gallbladder stones also present with choledocholithiasis. In individuals with acute calculous cholecystitis who are discharged without surgical intervention, the likelihood of subsequent gallstone-related complications increases progressively over time. The objective of the present study was to evaluate the safety, efficacy, and perioperative outcomes of performing single-session endoscopic retrograde cholangiopancreatography (ERCP) followed by laparoscopic cholecystectomy (LC) in the same operative setting. This combined approach, although less commonly adopted than the traditional two-session strategy, may provide meaningful benefits in the management of patients with concomitant gallbladder and common bile duct (CBD) stones.

Methods

A retrospective single-centre review from a tertiary care centre in South India was conducted between June 2014 and June 2024. Patients with gallbladder pathology and concomitant choledocholithiasis were evaluated and scheduled for ERCP and LC in the same session.

Results

A total of 147 patients were included in the study, with a median age of 49 years and a female predominance. Choledocholithiasis with cholelithiasis was the most common indication in the study. The majority of cases were cannulated within 10 minutes (51%) and in five or fewer attempts (95.9%). Stone extraction was successful in 136 cases (92.5%), with CBD clearance achieved in 130 cases. Open CBD exploration was performed in three cases, and laparoscopic CBD exploration was performed in one case. LC was performed during the same session.

Conclusion

Under careful patient selection, single-session ERCP plus LC will not only save time but also avoid repeated administration of anaesthesia. Therefore, whenever expertise is available, the single-session ERCP and LC strategy can be adopted.

## Introduction

Cholelithiasis, or gallstones, is seen in about 15% of the general population and can lead to a few complications, including cholecystitis, obstructive jaundice secondary to bile duct stones, and pancreatitis. Common bile duct (CBD) stones are seen in 10%-18% of patients with gallstones [1]. The current standard treatment for acute or chronic cholecystitis is laparoscopic cholecystectomy (LC) [2,3]. No single method or algorithm has been shown to be superior to others for treating patients with gallstones and concomitant CBD stones. Combining endoscopic retrograde cholangiopancreatography (ERCP) and LC into a single-step procedure is an evolving approach for the management of CBD stones (choledocholithiasis) and gallbladder disease.

This "one-step," or single-session, approach can offer several clinical advantages over the traditional two-step method, in which ERCP is performed before or after surgery in a separate session. This approach integrates intraoperative ERCP (IO-ERCP) with LC under the same anaesthesia. The advantages of the single-step procedure include reduced hospital stay and costs, faster recovery, reduced total procedure time, avoidance of delays between procedures, and a single exposure to anaesthesia. However, this approach has a few limitations, including the need for multidisciplinary coordination (surgery and gastroenterology), limited suitability in hospitals with infrastructure constraints, and reduced applicability in unstable patients or those with complex biliary anatomy. Current international guidelines consider both single-session and two-stage management to be appropriate treatment options for concomitant gallbladder and CBD stones, with the choice individualised according to patient factors, institutional resources, and available expertise.

Nevertheless, recent systematic reviews and meta-analyses [4,5] have demonstrated that the single-session approach achieves comparable technical success and safety while offering potential advantages, including reduced hospital stay, single anaesthesia exposure, and lower healthcare utilisation in appropriately selected patients. This study aimed to evaluate the safety, technical success, efficacy, and perioperative outcomes of single-session ERCP followed by LC in patients with concomitant cholelithiasis and choledocholithiasis.

## Materials and methods

A retrospective single-centre study of prospectively collected data from a tertiary care centre in South India was conducted between June 2014 and June 2024. Both ERCP and LC in this study were performed in a single surgical unit by the same operating surgeon within the same session. The patients excluded from the study included those aged below 18 years, patients with only gallbladder pathology or only CBD pathology, patients with conditions such as bile duct leak, bile duct injury secondary to operative procedures, gallbladder carcinoma, or cholangiocarcinoma, patients with an indication for ERCP for pancreatic disorders only, patients with previous upper gastrointestinal tract anastomosis, and patients with contraindications to general anaesthesia.

Suitability for the single-session approach was determined based on confirmation of both pathologies, fitness for general anaesthesia, and the treating surgical team's assessment that ERCP followed by LC could be safely completed during the same anaesthetic session. This study included only patients who underwent the planned single-session procedure. During the study period, 10 patients underwent ERCP followed by interval LC because of clinical or logistical considerations and were therefore excluded from the present analysis.

After obtaining informed written consent, all patients were clinically examined, evaluated with corresponding radiological investigations, and admitted for the proposed procedure. Diagnosis was confirmed using either abdominal ultrasonography, contrast-enhanced computed tomography of the abdomen and pelvis, or magnetic resonance cholangiopancreatography (MRCP). After pre-anaesthetic evaluation, patients were planned for single-session ERCP followed by LC. All procedures were done under general anaesthesia, initially in the prone position for ERCP and later in the supine position for LC. Preoperative diclofenac suppositories were administered per rectally to all patients to prevent post-ERCP pancreatitis. In all ERCPs, CBD cannulation was done with the help of flexible-tip guide wires, and biliary sphincterotomy was performed when required. Precut sphincterotomy was performed in difficult cannulation cases. Initial cholangiography was performed to delineate the biliary tract and identify the pathology involved. A check cholangiogram was performed to confirm the absence of further filling defects before stent placement. All LCs were performed in the supine position using standard four-port techniques, and the fifth port was placed in difficult cases when overdistended bowel obscured Calot’s dissection. Antegrade (fundus-first) gallbladder dissection was performed only when there was difficulty achieving the critical view of safety. Specimens were retrieved via the epigastric port in all cases. A drain was placed only in difficult cholecystectomies where bleeding or bile leak was anticipated. All procedures performed were recorded, and data were maintained prospectively over the years. This study aimed to evaluate the safety and efficacy of single-session ERCP with LC. All patients who underwent CBD stenting were advised to undergo stent removal after two to four weeks following normal postoperative check ultrasonography, MRCP (in selected cases), liver function tests, and complete blood count.

Patient data were collected from the case files, operative notes, and follow-up data with regard to immediate postoperative complications, residual stones, and recurrent stones. IBM SPSS Statistics for Windows, Version 23.0 (Released 2015; IBM Corp., Armonk, NY, USA), was used for data analysis; categorical data were represented as percentages and counts.

## Results

A total of 147 patients with concomitant gallbladder pathology and choledocholithiasis underwent single-session ERCP followed by LC during the study period. The mean age of the study population was 49 years, with a mean of 49.04 ± 16.25 years (range: 16-95 years), and there was a female predominance (95 patients, 64.6%). At least one comorbidity was present in 85 patients (57.8%), with diabetes mellitus being the most common, followed by hypertension (Table 1). The most frequent indication for intervention was choledocholithiasis with concomitant cholelithiasis, while a subset of patients also presented with cholangitis, CBD stricture, biliary pancreatitis, or acute cholecystitis. Abdominal pain was the most common presenting symptom, followed by nausea/vomiting, jaundice, and fever.

**Table 1:** Demographic and clinical characteristics of the study population (N = 147)

Characteristic	Value
Age (years), mean ± SD	49.04 ± 16.25
Age range (years)	16-95
Female sex	95 (64.6%)
Male sex	52 (35.4%)
Presence of comorbidity	85 (57.8%)
Diabetes mellitus	56 (38.1%)
Hypertension	38 (25.9%)
Ischemic heart disease	10 (6.8%)
Bronchial asthma	5 (3.4%)
Others	16 (10.9%)

Baseline biochemical and radiological parameters demonstrated a broad spectrum of disease severity, reflecting varying degrees of biliary obstruction and inflammation (Table 2). Leukocyte counts ranged from approximately 4,000 to over 24,000 cells/mm³, reflecting a broad spectrum of inflammatory response. Total serum bilirubin levels varied from 0.2 to 13.4 mg/dL, while direct bilirubin concentrations ranged from 0.1 to 10.4 mg/dL, indicating varying degrees of biliary obstruction. Similarly, serum alkaline phosphatase levels demonstrated marked heterogeneity, extending from values below 100 IU/L to levels exceeding 1,000 IU/L in select cases.

**Table 2:** Baseline clinical, biochemical and radiological characteristics Baseline laboratory and radiological evaluation revealed heterogeneous disease severity. Leukocyte count, bilirubin levels, alkaline phosphatase, CBD diameter, and stone size showed wide ranges, consistent with the varied clinical spectrum of choledocholithiasis and biliary obstruction. IHBR: intrahepatic biliary radicle; CBD: common bile duct

Variable	Mean ± SD or n (%)	Median	Range
Leukocyte count (cells/mm^3^)	10676.25 ± 3467.72	10160.00	4059.0-24098.0
Total bilirubin (mg/dL)	3.42 ± 2.24	3.20	0.2-13.4
Direct bilirubin (mg/dL)	2.07 ± 1.60	1.60	0.1-10.4
Alkaline phosphatase (IU/L)	226.29 ± 120.15	203.00	45.0-1042.0
CBD diameter (mm)	11.49 ± 3.61	11.20	3.6-22.0
Largest CBD stone size (mm)	9.09 ± 2.96	9.00	1.8-19.8
Cholangitis	24 (16.3%)	-	-
Biliary pancreatitis	6 (4.1%)	-	-
CBD stricture	24 (16.3%)	-	-
IHBR dilatation	128 (87.1%)	-	-
Single CBD stone	80 (54.4%)	-	-
Two CBD stones	35 (23.8%)	-	-
Three or more/multiple CBD stones	32 (21.8%)	-	-

Radiological evaluation revealed a wide distribution of CBD diameters, ranging from approximately 3.6 mm to over 22 mm, with the majority of patients exhibiting ductal dilatation consistent with obstructive pathology (Table 2). In terms of stone burden, most patients harboured a single CBD stone, while a notable subset had multiple stones. The maximum stone diameter also varied substantially, ranging from approximately 1.8 mm to 19.8 mm, encompassing both small and large calculi. Overall, the study population demonstrated substantial heterogeneity in clinical presentation, biochemical severity, ductal anatomy, and stone characteristics, underscoring the diverse spectrum of CBD stone disease encountered in patients undergoing ERCP.

**Figure 1:**
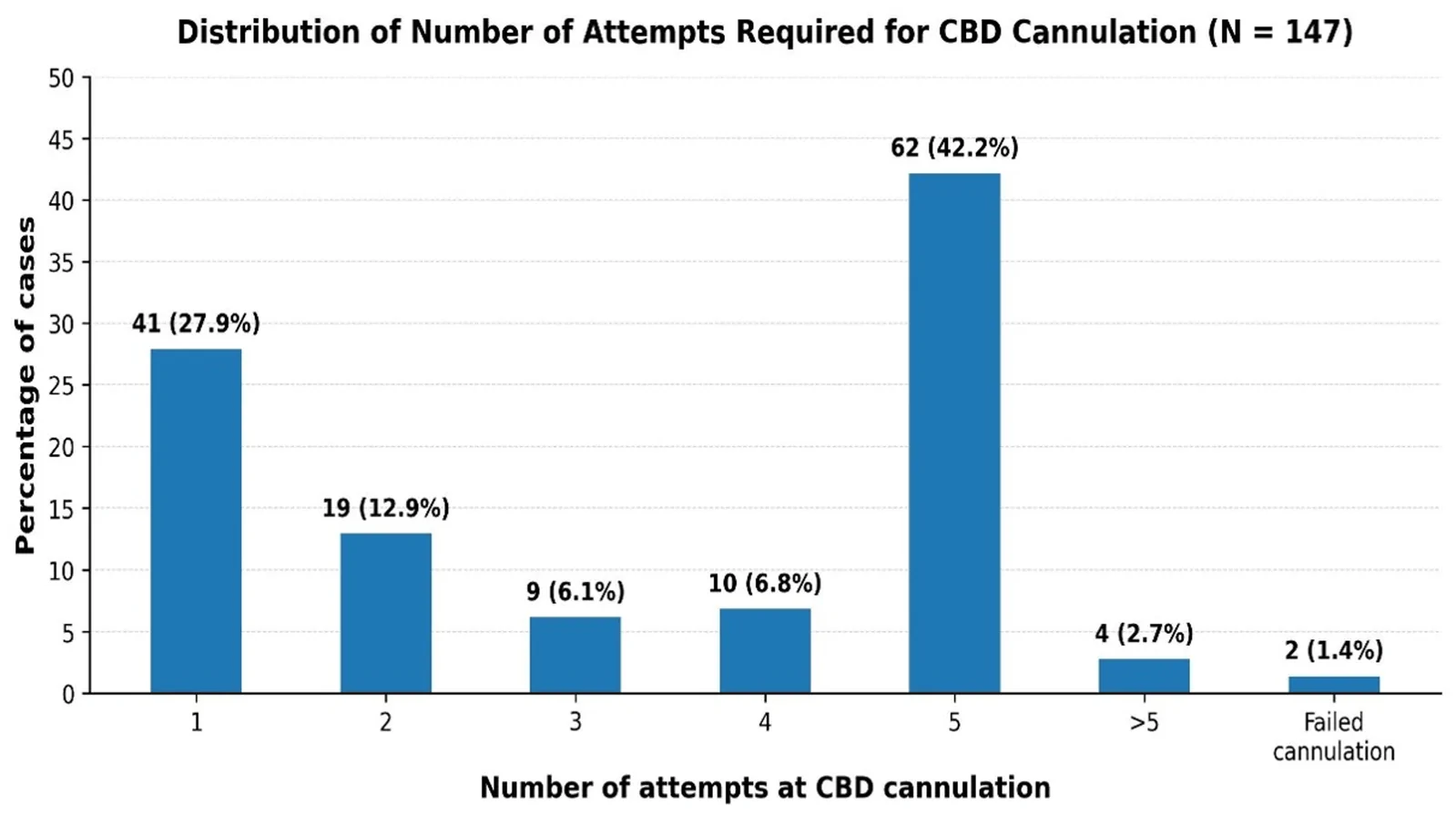
Distribution of number of attempts required for CBD cannulation among study participants (N = 147) Values are expressed as frequency, N (%) CBD: common bile duct

All patients first underwent ERCP followed by LC in the operative session. Standard biliary sphincterotomy was performed in 72 patients (49.0%), while precut sphincterotomy was required in 73 (49.7%). ERCP was deferred in two patients because of failed biliary cannulation (Table 3). Successful biliary cannulation was achieved within 10 minutes in 126 patients (86.9%) and within five or fewer attempts in 141 patients (95.9%) (Table 4 and Figure 1).

**Table 3:** Procedural characteristics and outcomes Stone extraction was achieved in 92.5% of patients, and complete CBD clearance in 88.4%. Biliary stenting was performed in most cases. Cholecystitis and adhesions were commonly observed intraoperatively, supporting the technical complexity of the combined single-session approach. CBD: common bile duct; ERCP: endoscopic retrograde cholangiopancreatography; LC: laparoscopic cholecystectomy

Variable	Value
Standard biliary sphincterotomy	72 (49.0%)
Precut sphincterotomy	73 (49.7%)
Failed bile duct cannulation	2 (1.4%)
Successful stone extraction	136 (92.5%)
Complete CBD clearance	130 (88.4%)
Biliary stenting	142 (96.6%)
Intraoperative CBD exploration	4 (2.7%)
Open CBD exploration	3 (2.0%)
Laparoscopic CBD exploration	1 (0.7%)
Mean ERCP duration (minutes)	33.7
Mean LC duration (minutes)	78.4
Intraoperative gallbladder status	
Normal gallbladder	24 (16.3%)
Cholecystitis	111 (75.5%)
Empyema	8 (5.4%)
Adhesions	84 (57.1%)

**Table 4:** Complete raw contingency data and association between clinical/procedural factors and successful stone extraction The values shown under Successful and Unsuccessful represent the raw contingency data used for analysis. Each p-value represents the overall association between the complete set of categories for that variable and the stone extraction outcome. Therefore, a single p-value is presented for each risk factor rather than separate p-values for individual rows. The p-values shown above are recalculated from the raw counts displayed in the table using the Pearson chi-square test. CBD: common bile duct; ERCP: endoscopic retrograde cholangiopancreatography

Risk factor	Category	Successful (n)	Unsuccessful (n)	Total (n)	Success rate (%)	Test used	χ² value	df	p-value
Age (years)	<=30	20	3	23	87.0	Pearson chi-square	3.382	5	0.641
	31-40	26	2	28	92.9				
	41-50	32	2	34	94.1				
	51-60	22	3	25	88.0				
	61-70	21	1	22	95.5				
	>70	15	0	15	100.0				
Time taken to cannulate CBD (minutes)	<5	48	4	52	92.3	Pearson chi-square	8.625	3	0.035
	5-9	71	3	74	95.9				
	10-14	10	1	11	90.9				
	>14	7	3	10	70.0				
Number of attempts to cannulate CBD	1	37	6	43	86.0	Pearson chi-square	19.133	5	0.002
	2	17	2	19	89.5				
	3	9	0	9	100.0				
	4	9	1	10	90.0				
	5	62	0	62	100.0				
	>5	2	2	4	50.0				
Duration of ERCP (minutes)	<=30	92	7	99	92.9	Pearson chi-square	1.679	3	0.642
	31-60	32	4	36	88.9				
	61-90	8	0	8	100.0				
	91-120	4	0	4	100.0				

**Figure 2:**
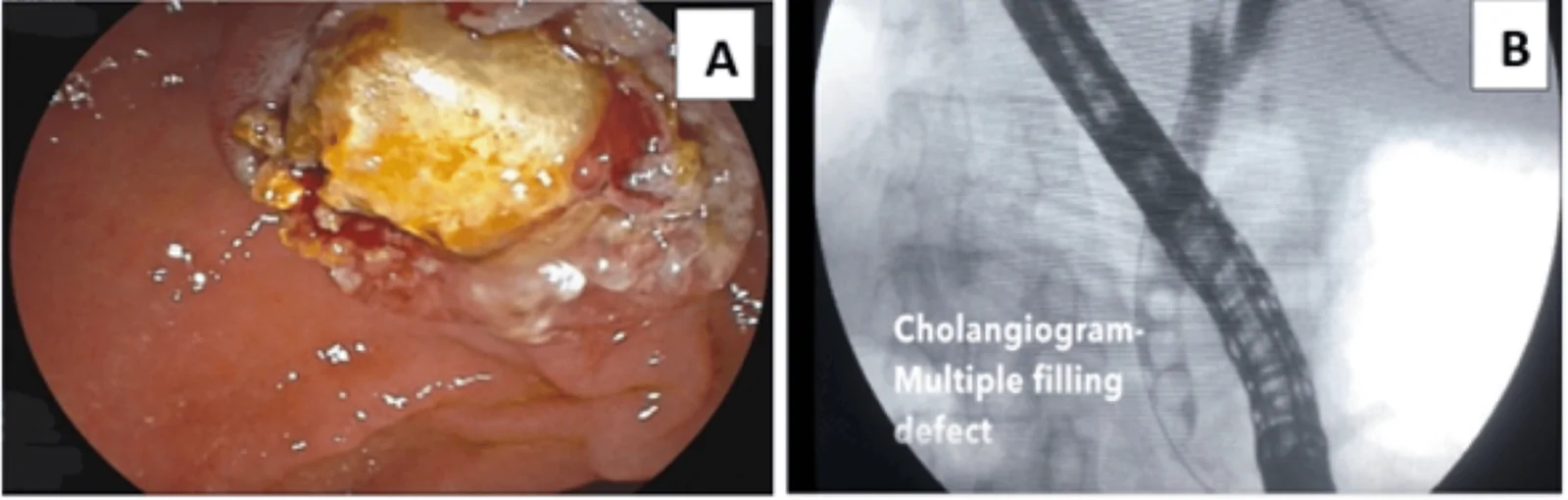
A large yellow coloured stone seen coming out of papilla (A) and cholangiogram during ERCP showed multiple filling defects in distal CBD (B) ERCP: endoscopic retrograde cholangiopancreatography

**Figure 3:**
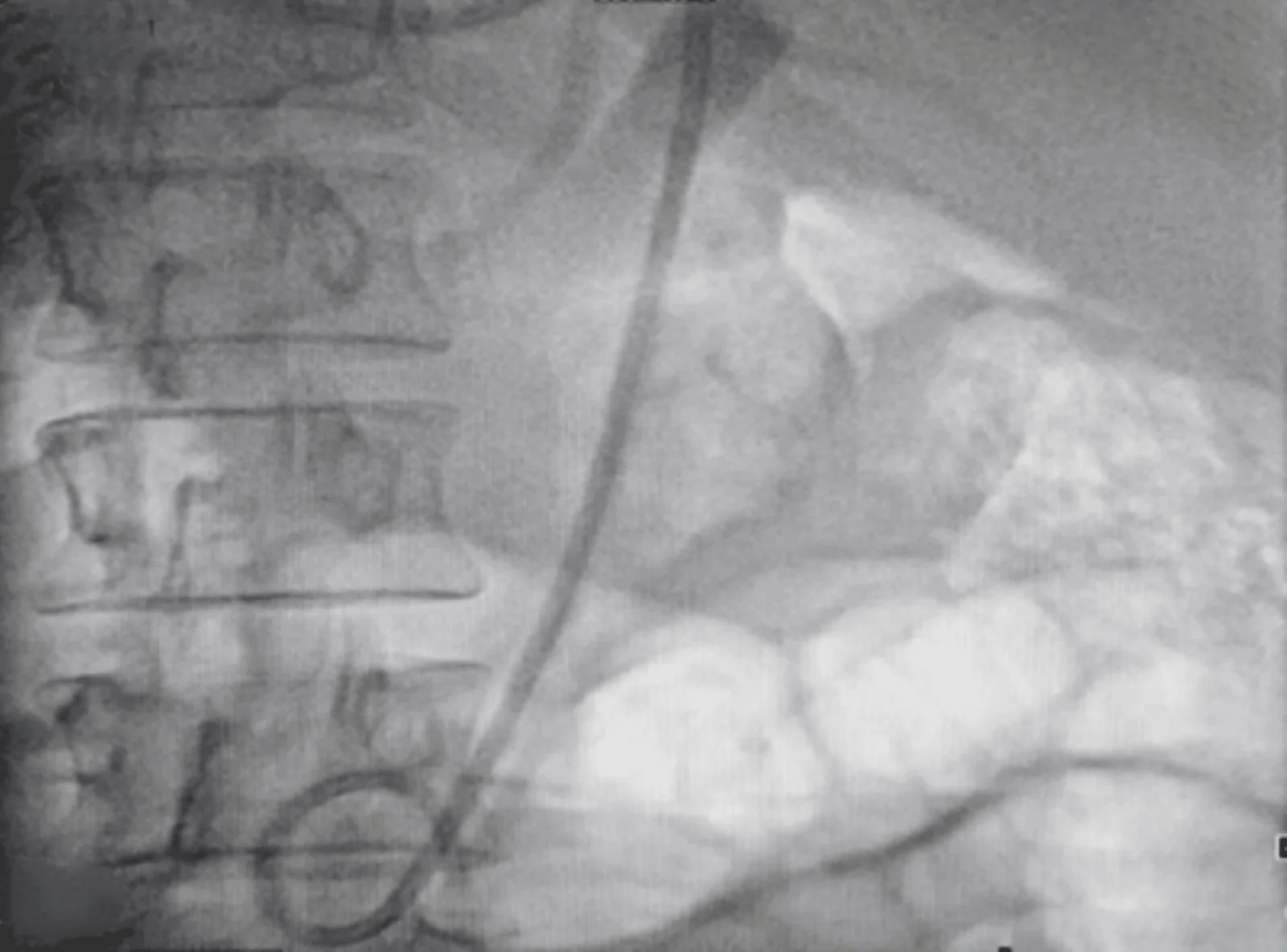
Fluoroscopy image showing CBD stent (double pig tail) insitu placed after stone extraction CBD: common bile duct

Successful stone extraction (Figure 2) was achieved in 136 patients (92.5%), and complete CBD clearance was achieved in the majority of patients (Table 3). Biliary stenting was performed in 142 patients (96%) (Figure 3). The mean ERCP duration was 33.7 minutes, and the mean operative time for LC was 78.43 minutes. Three patients underwent open CBD exploration, and one underwent laparoscopic CBD exploration.

LC was performed during the same session. Subtotal cholecystectomy was performed in two patients, and conversion to an open procedure was required in one case because of difficulty dissecting the gallbladder from the liver due to dense adhesions to the liver bed, along with dense omental adhesions (Table 3). Intraoperative findings included evidence of cholecystitis in 111 patients (75%), adhesions in 84 (57%), and empyema in 8 (5.4%) (Table 3).

The complete contingency data used to assess associations between clinical and procedural factors and successful stone extraction are presented in Table 4. A single p-value was calculated for each variable using all categories within that variable and stone extraction outcome. Age group was not significantly associated with successful stone extraction (χ² = 3.382, p = 0.641). However, both the time required for CBD cannulation (χ² = 8.625, p = 0.035) and the number of cannulation attempts (χ² = 19.133, p = 0.002) were significantly associated with stone extraction success. Increasing cannulation difficulty, reflected by prolonged cannulation time and a greater number of attempts, was associated with a higher likelihood of unsuccessful stone extraction. Duration of ERCP was not significantly associated with stone extraction success (χ² = 1.679, p = 0.642).

Procedure-related complications were uncommon (Table 5). One patient had postoperative bleeding of unclear aetiology, and another had sphincterotomy-site bleeding intraoperatively, which was controlled with a local injection of 1:10,000 adrenaline. Bile leak was seen in two cases secondary to partial (lateral) injury to the CBD, wherein difficult dissection of Calot's triangle was noted. The leak reduced over the course of two days, and the patients were managed conservatively. Three patients developed postoperative pancreatitis, as suggested by complaints of abdominal pain and elevated serum lipase levels. All the patients developed pancreatitis on POD 3 and were managed conservatively. Three patients (2%) died during the hospital course, with the causes attributed to aspiration pneumonia in one patient and septic shock in two; none of the deaths were procedure-related.

**Table 5:** Procedure-related complications and mortality ERCP: endoscopic retrograde cholangiopancreatography

Complication	N (%)
Sphincterotomy-site bleeding	1 (0.7%)
Perforation	1 (0.7%)
Post-ERCP pancreatitis	3 (2.0%)
Intraoperative bleeding	2 (1.4%)
Bile duct injury/bile leak	2 (1.4%)
In-hospital mortality	3 (2.0%)

## Discussion

Cholelithiasis is one of the most frequent biliary diseases, along with choledocholithiasis, in the Asian subcontinent, with a significant burden noted among the Indian population. The passage of gallstones from the cystic duct into the CBD is the most frequent cause of choledocholithiasis [6]. Nearly 18% of patients hospitalised for cholecystolithiasis have choledocholithiasis [2], and within the first year following cholecystectomy, approximately 3.8% will experience symptoms of choledocholithiasis [7], which emphasises the importance of concomitant management of both cholelithiasis and choledocholithiasis.

Previous studies have suggested that single-session ERCP followed by LC may offer several advantages over the traditional two-session approach, including single anaesthesia exposure, a shorter hospital stay, and lower postoperative complication rates, particularly post-ERCP pancreatitis. It also minimises residual stones and decreases the likelihood of requiring reoperation for stone extraction [8]. Furthermore, the one-step technique is considered superior because it reduces the preoperative waiting time [9] and eliminates the waiting interval between the two procedures.

In our present study, both ERCP and LC were performed by the same specialised surgeon in the same operative setting, adding the advantage of reducing, or completely eliminating, the switchover time between the two procedures and thereby reducing the overall operative time. These findings are consistent with the observations by ElGeidie et al., which showed that if ERCP and LC were performed by a single surgeon or a coordinated team, the operative time in the intra-ERCP+LC group would be relatively shorter [10]. Similar advantages were described by Easwaramoorthy et al., particularly in terms of improved communication and streamlined postoperative follow-up, which were also noted in our experience [11].

The procedural success and complication rates observed in our study are broadly consistent with those reported in previous literature, although direct comparisons should be interpreted cautiously because of differences in study design, patient populations, and disease severity. A meta-analysis by Tan et al. reported a choledocholithiasis clearance rate of 93.3% for the ERCP plus LC approach, which is comparable to the data observed in our study [12]. In addition to favourable technical success, the safety profile in our cohort was also comparable to that reported in the literature. Post-ERCP pancreatitis, one of the most common complications following ERCP, with a reported incidence of 1%-5% [13], occurred in 2% of our patients, which is consistent with the findings reported by Cotton et al. [14].

Beyond technical success and procedural safety, the single-session approach has been associated with several logistical and patient-centred benefits. Del Rio et al. reported improved patient acceptance of the one-step technique, attributing this to reduced hospitalisation and single anaesthesia exposure [15]. Similarly, Iodice et al. demonstrated the feasibility of the combined endoscopic-laparoscopic approach and reported a shorter hospital stay as an additional advantage [16]. Easwaramoorthy et al. further suggested that performing both procedures by the same surgeon may enhance continuity of care by facilitating communication and streamlining postoperative follow-up [11]. Collectively, these findings suggest that, in appropriately selected patients and experienced centres, the single-session approach may offer organisational and patient-centred benefits in addition to its technical feasibility.

The importance of timely surgical management is further underscored by data showing that, in patients discharged without surgery after an episode of acute calculous cholecystitis, the probability of recurrent gallstone-related events at 6 weeks, 12 weeks, and 1 year is 14%, 19%, and 29%, respectively [17]. Muhammedoğlu similarly concluded that single-stage management is associated with shorter hospitalisation, fewer imaging requirements, and particular benefit for patients with obstructive jaundice who may not tolerate a second anaesthetic exposure [17]. These observations are in keeping with our findings; however, direct comparison is limited by differences in study populations and the absence of a comparator group in the present study.

While our findings are generally in agreement with the published literature, these comparisons should be interpreted with caution. The present study was a retrospective, single-arm analysis without a comparator group, and variations in patient selection, baseline disease severity, procedural techniques, institutional protocols, and operator experience across studies may have influenced the reported outcomes. Therefore, our findings should be considered supportive of the feasibility and safety of the single-session approach within our study setting rather than evidence of superiority or equivalence to alternative management strategies.

Limitations

This study is limited by its retrospective, single-centre design, which may introduce selection bias and restrict generalisability. All procedures were performed by a single highly experienced surgeon, so the outcomes may not be applicable to centres with varying levels of expertise. The absence of a comparison group limits the ability to directly evaluate the superiority of the single-session approach.

## Conclusions

Single-session ERCP followed by LC appears to be a feasible and safe approach for the management of concomitant cholelithiasis and choledocholithiasis in carefully selected patients. In this retrospective single-centre study, the approach was associated with high rates of successful stone extraction and CBD clearance, along with low rates of conversion and procedure-related complications. However, in the absence of a comparator group, these findings should be interpreted as supporting the feasibility and safety of the single-session approach rather than demonstrating superiority over conventional two-stage management. Further prospective, multicentre comparative studies are warranted to validate these findings and assess their generalisability.
